# A comparison of the learning effects between TGfU-SE and TGfU on learning motivation, sport enjoyment, responsibility, and game performance in physical education

**DOI:** 10.3389/fpsyg.2023.1165064

**Published:** 2023-07-13

**Authors:** Yi-Hsiang Pan, Chen-Hui Huang, Wei-Ting Hsu

**Affiliations:** ^1^Graduate Institute of Physical Education, National Taiwan Sport University, Taoyuen, Taiwan; ^2^Department of Sport Promotion, National Taiwan Sport University, Taoyuen, Taiwan; ^3^Department of Physical Education and Kinesiology, National Dong Hwa University, Hualien, Taiwan

**Keywords:** motivation, responsibility, game performance, affective learning, physical activity

## Abstract

**Background:**

Both the Sport Education (SE) model and Teaching Games for Understanding (TGfU) have been connected to the theory of situated learning, which is a game-centered curricular model. TGfU emphasizes tactical awareness, decision making, and skill execution. The SE model provides a complete season during physical education (PE) lessons. Therefore, it is worth exploring the integration of TGfU with SE (TGfU-SE) model in PE courses, and whether the hybrid TGfU-SE model can achieve better learning effects for students than the TGfU model alone.

**Purpose:**

The purpose of the study was to compare the difference in learning effects between the TGfU-SE model and the TGfU model on students’ learning motivation, sport enjoyment, responsibility, and game performance.

**Methods:**

This study used a quasi-experimental design to compare different learning effects between the experimental group (TGfU-SE) and the control group (TGfU). The participants lived in Taiwan, including two junior high school PE teachers and four PE classes with a total of 90 students (TGfU-SE group, *n* = 46; TGfU group, *n* = 44). Each teacher taught two PE classes, one with an experimental group and one with a control group. This study used four research instruments, including the Responsibility Scale in Physical Education (RSPE), Learning Motivation Scale in Physical Education (LMSPE), Sport Enjoyment Scale in Physical Education (SESPE), and Game Performance assessment instrument (GPAI). Analysis of covariance (ANCOVA) and the independent *t*-test were used to analyze the data.

**Results:**

The results of this study showed that the TGfU-SE model had more positive learning effects on students’ learning motivation, sport enjoyment, responsibility, and game performance than the TGfU model.

**Conclusion:**

we concluded that the TGfU-SE model had a more positive influence on students’ learning performance than the TGfU model. It is suggested that the hybrid TGfU-SE model could be implemented effectively in the PE curriculum.

## Introduction

In the last few decades, some important learning theories in modern education, such as constructivism, situated learning, student-based learning, and cooperative learning, have been highlighted in PE curricula and teaching (Sidentop et al., 2004). In line with this trend, reforms have been moving towards a model-based curriculum for school PE in recent years (Lund and Tannehill, 2010; Hastie and Casey, 2014). Therefore, some scholars of sport pedagogy attempted to identify which model-based curriculum has better learning outcomes in the cognitive, psychomotor, and affective domains. Given the positive learning effects of the game-centered curriculum model in PE, they have gradually attracted widespread attention. The game-centered curriculum model encourages students to develop their game performance, which pertains to the cognitive and psychomotor domain, and to learning motivation, sport enjoyment, and responsibility, which refer to affective attitudes. Albuquerque et al. (2021) indicated that the small-sized games method was related to the positive development of practitioners, which was positively associated with goal setting and cognitive skills. Li et al. (2018) indicated that the term game-centered approaches has been gradually applied to teaching sports and games in PE lessons since the 1980s, including the Sport Education model and Teaching Games for Understanding. Generally, physical educators apply multiple models to conduct teaching activities in PE lessons and seldom use a single model to guide students to achieve learning goals.

Both SE and TGfU models are important models that transformed from teacher-centered to student-centered learning in PE (Farias et al., 2015). The theory of situated learning has been connected to SE and TGfU (Li et al., 2018, p. 360).

Situated learning theory, first initiated by Lave and Wenger (1991), states the skills and knowledge gained as a result of participating in a community of practice. Learning is fundamentally a social process and viewed as a situated activity that was called legitimate peripheral participation (LPP). LPP leads to individuals participating actively in the sociocultural practices of the community. SE and TGfU could provide more meaningful and purposeful content in authentic learning situations than other approaches (Dyson et al., 2004). The SE model has a structured season, but it lacks effective game strategies to improve student game performance. TGfU can improve tactical strategies, making up for the shortcomings of the SE model because TGfU connects tactics with skills in the learning context (Wang and Ha, 2013). Therefore, TGfU is integrated into the SE program to determine whether it will improve students’ learning performance. It is worth implementing the TGfU-SE hybrid model into PE lessons to obtain the results of empirical research.

### Teaching games for understanding

Bunker and Thorpe (1982) developed TGfU, a new teaching model that moves from tactics to skill and differs from traditional teaching in PE lessons. Students are guided to learn tactical strategies and motor skills through games, which could also enhance their learning motivation and game performance (Farias et al., 2015). Currently, problem-solving has become an important issue in the international PE curriculum (Wang and Ha, 2013). TGfU is intended to develop students’ competence in problem-solving in authentic game situations. Problem-based learning or inquiry learning is the central feature of developing an understanding to resolve problems (Harvey et al., 2018). Therefore, TGfU has become an important model for PE lessons in the past thirty years. TGfU is also an important course of PE teacher education in some countries (Stran et al., 2012). In empirical studies of TGfU, previous studies applied the action research approach as the main method to explore the implementation process and learning effect in PE lessons. The findings of these studies pointed out that this approach could obtain positive significant effects on students’ game performance (Harvey et al., 2010). In general, TGfU had better learning effects than the skill-oriented traditional teaching model for learning interest, tactical cognition, and game performance (Turner and Martinek, 1999; Harvey et al., 2010).

Some TGfU researchers have also developed innovative strategies to obtain improved learning performance, for example, integrating other curricula and teaching models into TGfU to obtain better learning effects. Hastie and Curtner-Smith (2006) conducted a study that integrated TGfU into SE courses and found that students improved significantly in game performance. Other researchers (Mesquita et al., 2012; Farias et al., 2015) integrated SE into a tactical game model that is similar to TGfU and found that students improved significantly in game performance and tactical awareness. The studies that integrated TGfU into SE courses were merely the pre-experimental design of the dual model, and it is impossible to understand how their effects differed from the learning effect of the TGfU model alone. Moreover, previous studies showed that TGfU could significantly improve students’ game performance (Turner and Martinek, 1999; Harvey et al., 2010). The current study follows previous studies (Hastie and Curtner-Smith, 2006; Mesquita et al., 2012; Farias et al., 2015) to determine whether there were different learning effects between the dual TGfU-SE model and the TGfU model alone.

In addition, Wang and Ha (2013) pointed out the potential advantages of the TGfU approach, including promoting the development of tactics and motor skills and enhancing learning motivation and responsibility, which enable students to experience enjoyment in sport situations. However, in empirical research, some studies have applied an objective measurement instrument, the Game Performance Assessment Instrument, to assess students’ tactical cognitive decisions and execution of motor skills in authentic situations. Mandigo et al. (2019) also indicated that TGfU could be effective in promoting some components of students’ development of physical literacy, including fitness skills and active participation.

Most studies have used mainly qualitative research to understand the development of students’ learning performance and lacked objective measurement instruments for the comprehensive evaluation of learning effects. Therefore, this study conducts a more comprehensive and objective assessment of students’ learning outcomes in addition to listing game performance as a dependent variable to examine additional learning effects, such as the dependent variables of responsibility, learning motivation, and sport enjoyment.

### Sport education model in physical education

There were five teaching models in the PE curriculum including the SE model, fitness education model, movement analysis model, developmental model, and personal meaning model (Jewett et al., 1995). Each model has its own characteristics, but there is no doubt that some researchers of sport pedagogy believe that the SE model is most aligned with contemporary educational views (Wallhead and O'Sullivan, 2005; Kinchin, 2006). In the 1980s, the traditional multi-activity model was the mainstream PE lesson in American primary and secondary schools (Sidentop et al., 2004). However, in this traditional multi-activity model, students spend 2 to 3 weeks on each unit of a course and are disengaged from authentic game situations, which often leaves them feeling bored and reduces their learning motivation. Therefore, Siedentop developed the SE model to reform the traditional teaching model in PE (Sidentop, 1994). The SE model, which aims mainly to educate students to be competent, literate, and enthusiastic sports participants, has six key characteristics: season, affiliation, formal competition, festivity, record keeping, and culminating event. The SE model is superior to the traditional multi-activity model because of its longer seasons and formal competitions, which provide the course with more structured content for students’ PE learning. All students are divided into various teams over the duration of the season, and formal competition provides a specific situation to build a team and move towards a common goal for the students throughout the season (Sidentop et al., 2004).

The SE model, a kind of situational learning, provides an authentic game experience that also enables students to feel more passionate about PE lessons (Kinchin, 2006). There is a learning atmosphere in the SE model that helps students participate actively in PE lessons (Tannehill and Lund, 2005). Compared with the traditional based-skill approach in PE, the SE course enables students to feel more learning passion when they are involved in PE lessons. This effect could improve students’ learning motivation and strengthen their willingness to participate in sports (Mohr et al., 2012; Perlman, 2012; Hastie et al., 2014; Parker and Curtner-Smith, 2014; Dyson et al., 2021; Teraoka et al., 2021). Mohr et al. (2012) conducted a study in which an SE course could improve students’ achievement of curricular goals and enhance their cooperation, teamwork and communication, and game performance. Méndez-Giménez et al. (2022) also indicated that the SE model could improve students’ self-determined motivation, basic psychological needs (BPN; competence, autonomy, and relatedness), and emotional intelligence. Manninen and Campbell (2022) indicated that the SE model could improve more need-supportive, promotes intrinsic motivation and prosocial attitudes more compared to the traditional instructional model in PE. The SE model involves a complete sport learning process in PE lessons that enables students to more comprehensively develop the cognitive, psychomotor, and affective domains (Wallhead and O'Sullivan, 2005; Pritchard et al., 2008; Hastie et al., 2011).

In the comparison of motivation between the SE model and the traditional skill-orientation model, a past study indicated that the SE model had a stronger learning effect than the traditional skill-orientation model in terms of sport enjoyment and interpersonal relationships for unmotivated students (Perlman, 2012). In the United States, only 32% of high school students continued to take elective PE courses each year after completing the required PE course, and students had low motivation to take elective PE courses. This situation has received much attention from PE researchers (Shen et al., 2010). Students’ learning motivation is generally low in the traditional teaching model, but the SE model can inspire students’ intrinsic motivation to participate in PE lessons (Perlman, 2012). The difference between the SE model and the traditional skill-orientation model is that SE courses arrange various formal competitions during the sports season, and students undergo a series of challenges to improve their learning performance. In terms of learning effects, SE courses improve students’ game performance and therefore are obviously better than the traditional teaching model (Pritchard et al., 2008). The SE model focuses on formal competition among teams, which could improve tactical awareness, strategic decision-making, and tactical execution. In fact, game performance is a combination of tactical strategy and skill execution in the context of the game, and this method is therefore a more objective approach to assessing the learning effect of sport performance. In summary, by designing challenging content, arranging formal competitions in PE courses, and allowing students to organize teams to compete with each other, teachers can enhance students’ learning performance through sports seasons.

### Integrating TGfU into sport education seasons

Games and sports are fundamental components of school PE curriculum and instruction. The importance of integrating both games and sports into PE content is reflected in the Society for Health and Physical Education K-12 grade-level outcomes in America, Canada, and Austria (Li et al., 2018). Both the SE model and TGfU have been connected to the theory of situated learning, which is a game-centered curricular model (Harvey et al., 2018). TGfU emphasizes that both tactical strategies and decision-making are important for students learning in PE lessons. Therefore, both tactical understanding and skill execution are emphasized in the TGfU context, as students can learn a variety of tactical strategies and motor skills in authentic game situations. The participants need a solid learning platform as a basis for developing their sport competence. SE courses provide a complete season during which students can apply tactics and skills that they have learned to real game situations. Therefore, students can obtain a complete learning process if TGfU is integrated into SE courses. If TGfU is combined with SE in a hybrid course, the following benefits could result. First, the SE model provides a good platform for games in the TGfU model. Students learn various tactical strategies and motor skills and need a formal competition platform to apply these new abilities in authentic game situations. The SE model has a formal competition season in which students can apply these tactics and motor skills in a real sport context. Second, students who are taught under the TGfU model alone learn only some tactics and motor skills in PE lessons but lack authentic games to provide a platform for them to practice these learned competencies. This kind of learning experience is a disjointed and incomplete learning process that lacks goal-oriented and structured content for students’ learning in PE lessons. Therefore, if the TGfU is integrated into SE courses, the hybrid TGfU-SE model will achieve better effects for students. In the past, some studies that integrated TGfU into SE courses, such as Hastie and Curtner-Smith (2006), Mesquita et al. (2012), Farias et al. (2015), and Gómez Buendía et al. (2022) were pre-experimental designs that could not examine whether the learning effects differed between the hybrid TGfU-SE model and the TGfU model alone. Therefore, it is necessary to compare the learning effects between the hybrid TGfU-SE model and the single TGfU model in PE lessons.

Hastie et al. (2013) conducted an empirical study of SE courses, and their results indicated that both team affiliation and formal competition were core components of the SE model. Therefore, the current study integrated TGfU into the SE curricular structure to determine how formal competition in the sports season influences the learning effect in TGfU lessons. This study adopted heterogeneous grouping in applying the TGfU model. The students were divided into different teams and cultivated team tactics and cooperative relationships within those teams. In addition, we integrated TGfU into the SE model as a TGfU-SE hybrid model that included all characteristics of the SE model, such as season, formal competition, festivity, record keeping, and culminating event, in addition to team affiliation. In short, the SE model creates a complete sports learning platform for students by providing formal competition throughout the sports season. Theoretically, the learning effects for students would be better in the TGfU-SE hybrid model than in the TGfU model alone. Incorporating the sports season would make the PE course more exciting from pre-season to post-season. However, we need to conduct empirical research to understand the different learning effects between the TGfU-SE hybrid model and the TGfU model alone.

### Purposes of this study

This study aimed to compare differences in learning effects between the hybrid TGfU-SE (experimental group) model and the TGfU model alone (control group) in terms of learning motivation, sport enjoyment, responsibility, and game performance. Based on the aforementioned literature concerning the SE season, a more positive influence effect on the TGfU program could be improved (Hastie et al., 2013). Accordingly, the proposed hypotheses of this study were as follows: the hybrid TGfU-SE model would improve learning effects on students’ learning motivation, sport enjoyment, responsibility, and game performance better than the TGfU model alone.

## Methods

### Research design

This study used quasi-experimental designs to compare the learning effects on learning motivation, sport enjoyment, responsibility, and game performance between the TGfU-SE hybrid model and the TGfU model alone.

The control group was based on the TGfU model. Each team was organized by heterogeneous grouping based on factors such as students’ motor skill ability and gender balance. The experimental group was based on the model of TGfU integrated into SE. Each team adopted heterogeneity, and the students were randomly assigned to the teams. Each student had his or her own fixed team for participating in any kind of activity and competition in the PE lessons. The TGfU-SE courses were designed, in addition to the TGfU components, to include six characteristics of the SE model: season, affiliation, formal competition, festivity, record keeping, and culminating event.

### Participants

This study was conducted over 10 weeks in junior high school PE lessons in Taiwan and included 2 qualified PE teachers and their students. Each teacher taught 2 classes, one experimental group and one control group, with a total of 90 students in the 4 classes (46 students in the experimental group, 24 boys and 22 girls, Mage = 15.02 ± 0.73 years, and 44 in the control group, 23 boys, and 21 girls, Mage = 14.78 ± 0.66 years). Two lessons of 45 min each per week made up the PE curriculum. This study adopted a counterbalance design in which each teacher taught both classes, one experimental group and one control group, to eliminate the influential factors associated with the teachers’ individual teaching ability that might have caused differences in students’ learning effects. This study was approved (IRB, C104059) by Institutional Review Board, FuJen Catholic University, Taiwan. Consent was obtained from both parents and students.

### Curriculum

#### Curricular plan

The curricular unit of the current study was basketball for 10 weeks with 20 PE lessons. After the preliminary design of the curricular plan, the curricular plans for the experimental TGfU-SE and control TGfU groups were both revised by two sport pedagogy scholars and five physical educators with teaching experience in TGfU and SE, ultimately leading to the design of the curricular program. The main check was to confirm whether the experimental and control groups of the curricular plan fit the characteristics of the TGfU and TGfU-SE models. The curricular plan was formalized after the revision. The curricular plan of the control group (TGfU) contained specific tactical awareness and skills-learning goals. Some modified games were designed for the course, tactical teaching discussions were conducted after learning activities, and random competition activities were included in each lesson (Table 1).

**Table 1:** TGfU curricular plan.

Lesson	Content	TGfU principle
1–2	Topic – rules and strategies for 3-on-3 game, Introduction and outline of the unit Beginning 1. Basic dribble, pass/catch the ball, shoot; 2. 3-on-3 basketball game	1. Create space to try to attack 2. Tactical understanding in games 3. Decision-making skills.
3–4	Topic – effective dribble games 1. Driving lay-up, one-on-one attack and defensedefense, two-on-two attack and defensedefense 2. 3-on-3 basketball game	1. Dribble and cut through to create space for an attack 2. Tactical understanding in games 3. Decision-making skills.
5–6	Topic – to win the competition 1. Offensive and defensive tactics 2. Pull-up jumper and driving to the hoop 3.3-on-3 basketball game	1. Allow teammates to cover and create a space to attack the basket 2. Tactical understanding in games 3. Decision-making skills.
7–8	Topic – limited-time delivery Offensive and defensive tactics 1. pass/fake moves, defensedefense/steals, 2. offensive and defensive tactics 3. 3-on-3 basketball game	1. Passing game, use fake moves to make lay-ups or pass opportunities 2. Tactical understanding in games 3. Decision-making skills.
9–10	Topic – basketball back-and-forth 1. offensive/defensive tactics 2. 3-on-3 basketball game	1. Passing the ball to a teammate 2. Tactical understanding in games 3. Decision-making skills.
11–12	Topic – shoot with great precision 1. Jump shot, driving to the hoop 2. 3-on-3 basketball game	1. Should I hold the ball or shoot at the goal? 2. Give and go, fast break 3. Tactical understanding in games 4. Decision-making skills.
13–14	Topic – making a feint to the east and attacking from the west 1. Defense/support/cover 2. 3-on-3 basketball game	1. Should I move towards the attacker or withdraw 2. Adjust the position of defensedefense 3. Tactical understanding in games 4. Decision-making skills.
15–16	Topic – shoot with great accuracy 1. Pick and roll 2. Driving to the hoop/catch and shoot 3. 3-on-3 basketball game	1. How should I place my body to protect ball possession? 2. Backdoor cut, give and go 3. Tactical understanding in games 4. Decision-making skills.
17–18	Topic – attack opponent’s field 1. Change defensedefense to attack 2. Defense/attack tactical application 3. 3-on-3 basketball game	1. Where should I shoot at? 2. Creating and defending space as a team. 3. Tactical understanding in games 4. Decision-making skills.
19–20	Semi-final for 3-on-3 basketball game 1. Final for 3-on-3 basketball game 2. Festivities	1. Develop team cooperation competence 2. Tactical understanding in games 3. Decision-making skills.

For the curricular plan of the experimental group (TGfU-SE), the TGfU course structure was integrated into the SE model to become the hybrid TGfU-SE program. The TGfU-SE teaching units contain both TGfU and SE courses, including teaching modified games of TGfU and six key features of the SE model: seasons, affiliation, formal competition, festivity, record keeping, and culminating event (Table 2).

**Table 2:** TGfU-SE curricular plan.

Season of SE	Lesson	Content	TGfU principle	Features of SE
Pre-season	1–2	Topic – rules and strategies for 3-on-3 game, Introduction and outline of the unit Beginning 1. Basic dribble, pass/catch the ball, shoot; 2. 3-on-3 basketball game 3. Introduction of team roles and responsibilities	1. Create space to try to attack 2. Tactical understanding in games 3. Decision-making skills.	Season Affiliation
	3–4	Topic – effective dribble games 1. Driving lay-up, one-on-one attack and defense, two-on-two attack and defense, 2. 3-on-3 basketball game	1. Dribble and cut through to create space for an attack 2. Tactical understanding in games 3. Decision-making skills.	Season affiliation
	5–6	Topic – to win the competition 1. Offensive and defensive tactics 2. Pull-up jumper and driving to the hoop 3. *Referee teaching/practice* 4.3-on-3 basketball game	1. Allow teammates to cover and create a space to attack the basket 2. Tactical understanding in games 3. Decision-making skills.	Season affiliation
	7–8	Topic – limited-time delivery Offensive and defensive tactics 1. pass/fake moves, defense/steals, 2. offensive and defensive tactics 3. *Referee teaching*/r*efereeing practice* 4. 3-on-3 basketball game	1. Passing game, use fake moves to make lay-ups or pass opportunities 2. Tactical understanding in games 3. Decision-making skills.	Season affiliation
	9–10	Topic – basketball back-and-forth 1. offensive/defensive tactics *2. Referee teaching/refereeing practice* 3. 3-on-3 basketball game	1. Passing the ball to a teammate 2. Tactical understanding in games 3. Decision-making skills.	Season affiliation
Season	11–12	Topic – shoot with great precision 1. Jump shot, driving to the hoop 2. Game design I. 3-on-3 basketball game	1. Should I hold the ball or shoot at the goal? 2. Give and go, fast break 3. Tactical understanding in games 4. Decision-making skills.	Season, affiliation, formal competition, record-keeping
	13–14	Topic – making a feint to the east and attacking from the west 1.Defense/support/cover 2. Game design II. 3-on-3 basketball game	1. Should I move towards the attacker or withdraw 2. Adjust position of defense 3. Tactical understanding in games 4. Decision-making skills.	Season, affiliation, formal competition, record-keeping
	15–16	Topic – shoot with great accuracy 1. Pick and roll 2. Driving to the hoop/catch and shoot 3. Game design III. 3-on-3 basketball game	1. How should I place my body to protect ball possession? 2. Backdoor cut, give and go 3. Tactical understanding in games 4. Decision-making skills.	Season, affiliation, formal competition, record-keeping
	17–18	Topic – attack opponent’s field 1. Change defense to attack 2. Defense/attack tactical application 3. Game design IV. 3-on-3 basketball game	1. Where should I shoot at? 2. Creating and defending space as a team. 3. Tactical understanding in games 4. Decision-making skills.	Season, affiliation, formal competition, record-keeping
Post-season	19–20	Semi-final for 3-on-3 basketball game 1. Final for 3-on-3 basketball game 2. Festivities	1. Develop team cooperation competence 2. Tactical understanding in games 3. Decision-making skill.	Season, affiliation, formal competition, record-keeping, culminating event, festivities

#### Model fidelity

The teaching activities of both groups were recorded by video. Two observers with 5 years of teaching experience analyzed the recordings to confirm the reliability of the two models. We designed a teaching behaviors checklist for TGfU and TGfU-SE based on Metzler’s model-based evaluation indicators (Metzler, 2011). The fit of Teacher A with TGfU was evaluated as 0.87 and 0.93, respectively, with a mean of 0.90. The fit of Teacher B with TGfU was evaluated as 0.89 and 0.85, respectively, with a mean of 0.87. The fit of Teacher A with TGfU-SE was 0.86 and 0.84, respectively, with a mean of 0.85, and the fit of Teacher B with TGfU-SE was 0.88 and 0.92, respectively, with a mean of 0.90. All of the means showed an acceptable level of reliability above 0.80 (Siedentop and Tannehill, 2000); therefore, the teaching behaviors of both teachers fit the fidelity rates of both models.

### Research instruments

This study applied 4 research instruments: the Responsibility Scale in Physical Education, Learning Motivation Scale in Physical Education, Sport Enjoyment Scale in Physical Education, and Game Performance assessment instrument.

### Responsibility scale in physical education

This study used the RSPE (Hsu et al., 2014) to assess students’ responsibility in PE lessons. Hsu et al. followed Hellison’s responsibility level to develop a reliability and valid responsibility scale for high school PE lessons. The RSPE has six components (effort, self-direction, following class rules, respect for others, helping others, and cooperation) with 26 items. The RSPE is valid and reliable for assessing students’ responsibility in high school PE lessons. The results of confirmatory factor analysis (CFA) indicated that all fit indicators achieved the measurement criteria (*χ^2^* = 617.82, df = 293, *p* < 0.05; TLI = 0.90; CFI = 0.91; RMSEA = 0.06; SRMR = 0.06); the basic criteria of composite reliability (0.87, 0.82, 0.82, 0.84, 0.86, 0.84) and average variance extracted (0.63, 0.53, 0.49, 0.57, 0.55, 0.57) were also achieved.

### Learning motivation scale in physical education

The LMSPE was developed by Pan (2014) based on Bandura’s conceptualization of self-efficacy in social cognitive theory and focusing on students’ motivation factors in high school PE lessons. This learning motivation scale has four components: value, expectation, affective, and self-efficacy. The value component refers to students’ viewpoints concerning why they engage in a PE curriculum. The expectation component refers to students’ beliefs regarding whether they can accomplish a task in PE. The affective component refers to students’ feelings regarding what is required to exhibit a positive attitude in PE. The self-efficacy component refers to students’ beliefs concerning their ability to successfully perform physical activities. The examination of the LMSPE reliability and validity achieved the fit criteria, and the CFA results showed acceptable fit (χ2 (98) = 298, *p* < 0.05; RMSEA = 0.07, GFI = 0.90, CFI = 0.97). In the LMSRE, the average variance extracted was 0.64, 0.58, 0.73, and 0.72, and the composite reliability was 0.88, 0.84, 0.86, and 0.91. Each item was rated by a six-point Likert-type scale that ranged from 6 (strongly agree) to 1 (strongly disagree). The LMSPE is both a reliability and validity measurement instrument that includes four factors and 16 items.

### Sport enjoyment scale in physical education (SESPE)

The SESPE was developed by Lin et al. (2016) based on both Scanlan and Lewthwaite’s sport enjoyment model and Garn and Cothran’s (2006) Fun Factor Scale in Physical Education. The SESPE has reliability and validity for the Chinese version of Garn and Corthan’s scale. The results of exploratory factor analysis (EFA) and CFA showed that the Chinese version of the SESPE had 4 factors with 15 items and achieved acceptable goodness-of-fit criteria (*χ*^2^ = 208.27, χ2/df = 2.47, GFI = 0.90, NFI = 0.97, NNFI = 0.97, CFI = 0.97, RFI = 0.96, IFI = 0.97, RMSEA = 0.09). The Chinese version of the SEPE had good reliability and validity and could be used in high school PE lessons. Scanlan and Lewthwaite (1986, p. 33) defined four-factor components: 1. achievement – intrinsic refers to predictors related to personal perceptions of competence and control, such as the attainment of mastery goals and perceived ability; 2. achievement – extrinsic refers to predictors related to personal perceptions of competence and control that are derived from other people, such as positive social evaluation and social recognition of achievement; 3. non-achievement – intrinsic refers to predictors related to physical activity and movement, such as sensations, tension, action, exhilaration, and competition, such as excitement; and 4. non-achievement – extrinsic refers to predictors related to non-performance aspects of sport, such as affiliation with peers and having positive interactions with teachers/adults involved in the experience.

### Game performance assessment instrument

The GPAI is a reliable and valid method for assessing game performance. This study used the GPAI developed by Mitchell et al. (2006), which includes 3 elements: decision-making, skill execution, and support. Decision-making refers to making appropriate decisions about what to do, for example, with the ball during a game; skills execution refers to the efficient execution of selected skills; and support refers to the provision of appropriate support for a teammate who has the ball by being in a position to receive a pass. The three elements were applied to assess students’ game performance. Memmert and Harvey (2008) indicated that GPAI has good validity and reliability based on the research findings of Oslin et al. (1998) as follows: (a) The observer reliability of the interobserver agreement measures was very high (>0.80). (b) The reliability of the GPAI components, the test–retest was used. The stability-reliability coefficients reached the acceptance level (>0.80). (c) The validity of the GPAI contained both content validity and construct validity. According to the construct validity, in 66% of the cases, the results of the GPAI components can be distinguished between students ranked high or low in-game.

### Data analysis

This statistical analysis method was used to examine differences between the experimental and control groups in terms of game performance, responsibility, learning motivation, and sport enjoyment. To compare the learning effects between the TGfU-SE experimental group and the TGfU control group, first, this study tested the homogeneity of within-group regression coefficients. If the within-group regression coefficients were homogeneous, a One-way analysis of covariance (ANCOVA) was performed to compare the differences in learning effects between the experimental group and the control group. If the within-group regression coefficients were heterogeneous, an independent t-test was used to conduct the progress score between the pre-test and post-test scores for these independent variables. When an ANCOVA was conducted, the Cohen η^2^ value was used to estimate the effect size (Cohen, 1988), with η^2^ ≥ 0.14 indicating a large effect size, 0.14 > η^2^ ≥ 0.06 indicating a medium effect size, and 0.06 > η^2^ indicating a small effect size. When an independent *t*-test was used, Cohen’s d value was used to estimate the effect size, with d = 0.80 indicating a large effect size, d = 0.50 indicating a medium effect size, and d = 0.20 indicating a small effect size. The significance level for this study was set at α = 0.05.

## Results

### Comparing the learning effect between TGfU-SE and TGfU

To determine the differences in learning effects between the TGfU-SE experimental group and the TGfU control group, Table 3 presents the means and standard deviations of the pre-and post-test scores for the two groups for sports enjoyment, learning motivation, responsibility, and game performance.

**Table 3:** ANCOVA for the TGfU and TGfU–SE groups on motivation, enjoyment, responsibility, and game performance.

Components	Group	*N*	Pre-test		Post-test		ANCOVA		Effect Size η^2^
			*M*	SD	*M*	SD	*F*	*p*	
Motivation									
Value	TGfU	46	5.43	0.82	5.26	1.13			
	TGfU-SE	44	5.40	0.87	5.55	0.80			
Expect	TGfU	46	5.18	0.99	5.03	1.20			
	TGfU-SE	44	5.31	0.94	5.56	0.66			
Self-efficacy	TGfU	46	4.73	0.86	4.70	1.16	6.12^*^	0.015	0.07
	TGfU-SE	44	4.62	1.08	5.05	0.98			
interest	TGfU	46	5.12	0.86	5.04	1.13	11.67^*^	0.001	0.12
	TGfU-SE	44	5.14	0.87	5.48	0.85			
Enjoyment									
Achievement-Intrinsic	TGfU	46	4.86	1.14	4.94	1.16	3.97^*^	0.048	0.05
	TGfU-SE	44	4.99	0.87	5.32	0.79			
Achievement-Extrinsic	TGfU	46	5.17	0.95	5.07	1.16			
	TGfU-SE	44	5.23	0.89	5.52	0.61			
Nonachievement-Intrinsic	TGfU	46	4.92	1.12	4.92	1.08	5.66^*^	0.020	0.06
	TGfU-SE	44	5.04	0.93	5.30	0.86			
Nonachievement-Extrinsic	TGfU	46	4.69	1.12	4.73	1.11	6.29^*^	0.014	0.07
	TGfU-SE	44	4.73	0.91	5.10	0.87			
Responsibility									
Effort	TGfU	46	4.91	0.92	4.89	1.08	6.93^*^	0.010	0.08
	TGfU-SE	44	5.13	0.73	5.43	0.62			
Self-direction	TGfU	46	4.66	1.08	4.67	1.15	9.35^*^	0.003	0.10
	TGfU-SE	44	4.86	0.85	5.28	0.77			
Following class rule	TGfU	46	5.63	0.57	5.43	1.06			
	TGfU-SE	44	5.55	0.55	5.70	0.45			
Respect others	TGfU	46	4.86	0.88	4.92	1.21			
	TGfU-SE	44	5.07	0.79	5.39	0.58			
Help others	TGfU	46	4.56	0.92	4.59	1.12	9.52^*^	0.003	0.10
	TGfU-SE	44	4.80	0.82	5.23	0.68			
Cooperation	TGfU	46	4.72	1.04	4.84	1.10			
	TGfU-SE	44	5.13	0.85	5.39	0.69			
Game performance									
Decision making	TGfU	46	47.17	20.01	50.78	19.83	5.46^*^	0.022	0.06
	TGfU-SE	44	45.52	22.58	58.50	15.56			
Skill execution	TGfU	46	38.93	20.99	48.39	15.22			
	TGfU-SE	44	37.02	19.99	50.34	13.71			
Support	TGfU	46	43.13	17.98	54.50	22.00			
	TGfU-SE	44	46.30	24.73	60.23	18.22			

*p < 0.05.

In terms of learning motivation, the homogeneity of within-group regression coefficients was tested, and the results for self-efficacy (*F*_(1,86)_ = 3.77, *p* > 0.05) and interest (*F*_(1,85)_ = 3.53, p > 0.05) implied that the above two components of learning motivation were homogeneous therefore ANCOVA was performed. As presented in Table 3, in the univariate analysis of covariance, there were differences between the two teaching models regarding the self-efficacy (*F*_(1,87)_ = 6.12, *p* < 0.05) and interest (*F*_(1,86)_ = 11.67, *p* < 0.05) components of learning motivation, with the TGfU-SE group performing better than the TGfU group. The effect size of self-efficacy was medium (η ^2^ = 0.07), and the effect size of interest was also medium (η ^2^ = 0.12). This study used an independent *t*-test to conduct statistical analysis for the progress score between the pre-test and post-test scores for these independent variables the value (*F*_(1,86)_ = 12.15, *p* < 0.05) and expectation (*F*_(1,85)_ = 10.77, *p* < 0.05) components because the results for implied that the within-group regression coefficients were heterogeneous. As presented in Table 4, the TGfU-SE experimental group showed more significant progress than the TGfU control group, including a significant difference in value (*t*_(88)_ = 3.50, *p* < 0.05), with a medium effect size (*d* = 0.73), and a significant difference in expectation (*t*_(88)_ = 2.48, *p* < 0.05), with a medium effect size (*d* = 0.53) in learning motivation.

**Table 4:** Independent *t*-test for progress score for TGfU and TGfU-SE group.

Components	TGfU(n = 44)		TGfU-SE(n = 46)		Independent t-test		Effect size Cohen’s *d*
	Mean	SD	Mean	SD	*t-valve*	*p valve*	
Motivation							
Value	−0.38	0.73	0.16	0.74	3.50^*^	0.001	0.73
Expect	−0.15	0.77	0.25	0.75	2.48^*^	0.015	0.53
Enjoyment							
Achievement-Extrinsic	−0.10	0.64	0.29	0.73	2.70^*^	0.008	0.55
Responsibility							
Following class rule	−0.21	0.67	0.15	0.51	2.83^*^	0.006	0.60
Respect others	0.06	0.83	0.32	0.72	1.60	0.113	
Cooperation	0.13	0.72	0.27	0.75	0.92	0.362	
Game performance							
Support	11.37	14.48	13.93	21.07	0.67	0.51	

^*^p < 0.05.

In terms of sport enjoyment, the homogeneity of within-group regression coefficients was tested, and the results for achievement – intrinsic (*F*_(1,84)_ = 3.49, *p* > 0.05) non-achievement – intrinsic (*F*_(1,85)_ = 1.18, *p* > 0.05) and non-achievement – extrinsic (*F*_(1,86)_ = 0.72, p > 0.05) implied that the within-group regression coefficients of these 3 components of sport enjoyment were homogeneous; therefore, ANCOVA was performed in the univariate analysis of covariance, as presented in Table 3, indicating differences between the two teaching models regarding achievement – intrinsic (*F*_(1,85)_ = 3.97, *p* < 0.05) and non-achievement – intrinsic for interest (*F*_(1,86)_ = 5.66, *p* < 0.05) and non-achievement – extrinsic (*F*_(1,87)_ = 6.29, *p* < 0.05) for sport enjoyment, with the TGfU-SE group showing better results than the TGfU group. The effect size of achievement – intrinsic was small (η^2^ = 0.05), that of non-achievement – intrinsic was medium (η^2^ = 0.06) and that of non-achievement – extrinsic was medium (η^2^ = 0.07). However, the achievement – extrinsic results (F _(1,86)_ = 21.75, *p* < 0.05) implied that the within-group regression coefficients were heterogeneous. Therefore, we used an independent t-test to conduct statistical analysis for the progress score between the pre-test and post-test scores for these independent variables. As presented in Table 4, the statistical analysis results indicated that the TGfU-SE experimental group made more significant progress than the TGfU control group in achievement – extrinsic (*t*_(88)_ = 2.70, *p* < 0.05) with a medium effect size (*d* = 0.55).

In terms of responsibility, the homogeneity of within-group regression coefficients was tested, and the results for three components of responsibility, effort (*F*_(1,85)_ = 2.63, *p* > 0.05), self-direction (*F*_(1,86)_ = 2.99, *p* > 0.05), and help others (*F*_(1,86)_ = 0.38, *p* > 0.05), implied that the within-group regression coefficients were homogeneous. Therefore, ANCOVA was performed in the univariate analysis of covariance, As presented in Table 3, indicating differences between the two teaching models regarding effort (*F*_(1,86)_ = 6.93, *p* < 0.05), self-direction (*F*_(1,87)_ = 9.35, p < 0.05) and help others (*F*_(1,87)_ = 9.52, *p* < 0.05), with the TGfU-SE group showing better results than the TGfU group. Effort had a medium effect size (η ^2^ = 0.08), self-direction had a medium effect size (η ^2^ = 0.10) and help others had a medium effect size (η ^2^ = 0.10). However, the results for following class rules (*F*_(1,86)_ = 33.25, *p* < 0.05), respect for others (*F*_(1,85)_ = 12.91, *p* < 0.05), and cooperation (*F*_(1,86)_ = 6.45, *p* < 0.05) implied that the within-group regression coefficients were heterogeneous. Therefore, we used an independent t-test to conduct statistical analysis for the progress score between the pre-test and post-test scores for these independent variables. As presented in Table 4, the statistical analysis results indicated that the TGfU-SE experimental group had made more significant progress than the TGfU control group in following class rules (*t*_(88)_ = 2.83, *p* < 0.05), with a medium effect size (*d* = 0.60), but both respect for others (*t*_(88)_ = 1.60, *p* > 0.05) and cooperation (*t*_(88)_ = 0.11, *p* > 0.05) were not significantly different between the TGfU-SE model and TGfU model.

In terms of game performance, the homogeneity of within-group regression coefficients was tested, and the results showed that decision-making (*F*_(1,86)_ = 0.58, p > 0.05) and self-direction (*F*_(1,86)_ = 1.96, *p* > 0.05) were homogeneous, implying that the within-group regression coefficients were homogeneous. Therefore, ANCOVA was performed in the univariate analysis of covariance, as presented in Table 3, indicating differences between the two teaching models regarding decision-making (*F*_(1,87)_ = 5.46, *p* < 0.05), with the TGfU-SE group showing better results than the TGfU group and effort with a medium effect size (η ^2^ = 0.06); however, there was no significant difference in skill execution (*F*_(1,87)_ = 1.15, *p* > 0.05). The results for support (*F*_(1,86)_ = 11.17, *p* < 0.05) implied that the within-group regression coefficients were heterogeneous. Therefore, we used an independent t-test to conduct statistical analysis for the progress score between the pre-test and post-test scores for this independent variable. As presented in Table 4, the statistical analysis results indicated that support (*t*_(88)_ = 0.51, *p* > 0.05) was not significantly different between the TGfU-SE and TGfU groups.

## Discussion

The purposes of the study were to compare the differences in learning effects on students’ sport enjoyment, learning motivation, responsibility, and game performance between the TGfU-SE and TGfU models. The results of this study showed that the TGfU-SE model of the experimental group had more positive learning effects on students’ game performance (including the decision-making component), responsibility (including the components of effort, self-direction, helping others, and following class rules), learning motivation (including the components of value, expectation, self-efficacy, and interest); and sport enjoyment (including achievement–intrinsic, achievement–extrinsic, non-achievement–intrinsic, and non-achievement–extrinsic) than the TGfU model of the control group. Linking findings to previous research, the TGfU-SE model could enhance game performance (Hastie and Curtner-Smith, 2006; Mesquita et al., 2012; Farias et al., 2015). It is also consistent with previous studies; Gómez Buendía et al. (2022) showed that the hybrid TGfU-SE model could generate positive effects on students’ enjoyment and sportsmanship. Gil-Arias et al. (2017) presented that the hybrid TGfU-SE model did have a significant positive influence on the satisfaction of the autonomy and competence components. In the situated learning theoretical framework for teaching in PE, both the SE model and TGfU are game-centered approaches based on situated learning theory (Li et al., 2018). The experimental group integrated the TGfU model into the SE model. The TGfU model emphasized that both game strategy and skill execution are important in learning contexts. In the TGfU model, students make decisions and execute skills to develop good game performance. Therefore, PE teachers need to provide authentic game situations to develop students’ sport competence, including game strategies and motor skills. The SE model provides a complete season to allow students to apply what they have learned to strategies and motor skills in real-game situations.

In a hybrid TGfU-SE model, in terms of game performance, students could learn more tactical awareness and skill execution in authentic situations. Students with low skill levels want to participate in teams because they enjoy having a team affiliation. This meant that students would pay attention to their own learning to improve their game performance. In past studies, TGfU was integrated with the SE model in a hybrid model that enhanced game performance, and the results were similar to those of past studies (Hastie and Curtner-Smith, 2006; Mesquita et al., 2012; Farias et al., 2015). TGfU-SE model can enable students to learn game strategies and skill execution across game seasons in the SE model. Students continue to learn tactical strategies and practice skill execution over a season so that they can enhance their game performance over time. The TGfU model guides students to appreciate games, understand tactical awareness, make decisions, and execute skills, ultimately achieving good game performance. TGfU model also emphasizes tactical understanding and skill execution. Past studies have shown that TGfU can significantly improve students’ game performance (Turner and Martinek, 1999; Harvey et al., 2010). The results of the study were consistent with the research hypothesis and verified Farias et al. (2015) suggestion that a hybrid sport education-invasion game competence model could promote improvements in students’ game performance and understanding. The SE model provided an authentic learning environment, and the TGfU provided learning tasks focused on tactical content and skill execution. Some studies have indicated that the SE model can improve learning effects in learning motivation, sport enjoyment, responsibility, and game performance. The results also verify Farias et al.‘s (2015) finding that the SE model can significantly improve students’ game performance and Wang and Ha’s (2013) finding that TGfU can improve students’ game performance. Therefore, integrating TGfU with SE can enable students to improve game performance in the hybrid TGfU-SE model. Li et al. (2018) indicated that situated game teaching through set plays (SGTSP) has the potential to enhance curricular development and teach tactical decision-making in games in PE. TGfU, SE, and SGTSP are game-centered approaches to teaching sports and games for school PE in an authentic setting, which can improve students’ game performance in PE lessons.

In terms of responsible behavior, when TGfU was integrated into the SE model, students can foster teamwork and perform responsible behavior during the season in the SE context, which is in line with Sidentop et al.‘s (2004) finding that the SE model can develop students’ positive affective behaviors. Some key findings from Wallhead and O'Sullivan (2005) indicated that SE increases the level of interaction and cooperation between students, and teachers perceived the model as fostering leadership, teamwork, peer support, and active pursuit of socially responsible and equitable participation. The SE model includes a longer season and formal competitions as well as a more structured learning process and group affiliation to promote teamwork learning and social interaction in PE lessons. A study conducted by Mohr et al. (2012) indicated that the SE model can effectively assist students in achieving curricular goals and enhance teamwork and mutual trust among members of a group. The current study confirmed that the hybrid TGfU-SE model can significantly improve students’ responsible behavior. Through seasons of SE, students’ personal and social responsibility can be developed, including behaviors such as cooperation, self-direction, respect for others, effort, and helping others. The SE model can provide students with responsibility for various roles in their teams (Li et al., 2018). Therefore, integrating TGfU with the SE program can improve students’ responsible behavior.

In terms of learning motivation, when TGfU was integrated into the SE model, students’ learning motivation was also enhanced in the learning process. Research findings on the SE model have suggested consistent results regarding students’ enhanced enthusiasm and motivation (Wallhead and O'Sullivan, 2005; Hastie et al., 2011). Some previous studies have indicated that the SE model provides an authentic game situation that promotes students’ motivation to learn and strengthens their willingness to participate in sports contexts (Mohr et al., 2012; Hastie et al., 2014; Parker and Curtner-Smith, 2014). Research grounded on motivational theories has shown positive changes when students participate in the SE model, which also provides unmotivated students with an increased opportunity to engage in higher levels of physical activity (Perlman, 2012). Therefore, integrating TGfU with the SE model can enhance students’ learning motivation more than the TGfU model alone.

In terms of sport enjoyment, both the TGfU and SE models can promote learning enjoyment in PE lessons. Through reflective thinking and problem-solving strategies in the TGfU model, students gain team cohesion through the season of the SE model. Sport enjoyment was also achieved virtually and verified Perlman’s (2012) finding that the SE model has a significant positive effect on students’ sense of pleasure. Garn and Cothran (2006, p. 284) indicated several conclusions concerning sport enjoyment. First, there are many sources that can make sports enjoyable, depending on the individual (e.g., friendships, mastery experiences, recognition, and movement sensation), and a variety of sources should be made available to ensure an enjoyable experience. Second, intrinsic and extrinsic factors of achievement can be enjoyable. One can experience enjoyment in sports from feeling competent while performing the task (i.e., intrinsic motivation) as well as receiving recognition from an outside source after its successful competition (i.e., extrinsic motivation). Third, non-achievement factors in sports, such as socializing with friends, can enhance enjoyment and achievement. Hastie et al. (2011) pointed out that an SE program can form a learning platform that enables students to acquire more pleasure in participation, games, and interacting with peers. Therefore, integrating TGfU into an SE model enables students to obtain more enjoyment from PE lessons.

Three core issues are related to the developmental tendency of PE curricula in America, England, and China (Wang and Ha, 2013): (1) three domains (i.e., psychomotor, cognitive, and affective) are incorporated; (2) PE curricula in most countries are related to social and interpersonal behaviors; and (3) problem solving, etc., issues have become major concerns of PE curricula. Models-Based Practice (MBP) is an important approach to improve students’ learning effects in these domains (e.g., affective, cognitive, psychomotor). The popularized notion of MBP is one that focuses on the delivery of a model, e.g., cooperative learning, sports education, teaching personal and social responsibility, and teaching games for understanding. Indeed, while an abundance of research studies have examined the delivery of a single model and some have explored hybrid models, few have sought to meaningfully and purposefully connect different models in a school’s curriculum (Casey and MacPhail, 2018, p. 294). In the current study, the findings showed that the hybrid TGfU-SE model had a stronger learning effect on learning motivation, sport enjoyment, responsibility, and game performance than the TGfU model alone. Therefore, PE teachers could integrate TGfU into the SE model to form a hybrid TGfU-SE model so that the TGfU model would involve formal competition over the SE season to promote better learning effects in PE lessons. Although most of the results support the research hypotheses, the study needs to be replicated on much larger samples and in conjunction with control groups to confirm the learning effects of the TGfU-SE model. However, this study has a number of limitations. First, the research used *purposive sampling* to obtain the samples and only two PE teachers and their students participated in this study. Therefore, there is a limiting factor for the generalization of research findings. This small number of teachers make it difficult to generalize the results to other teachers who complement these pedagogical model in PE. These kinds of studies will have more empirical evidence to confirm the research findings through more teachers participating in this hybrid model of the PE program. Second, this study was conducted over only 10, comprising 20 PE lessons, which means it could not promote more positive learning effects over such a short-term course. Therefore, it is a better research design to extend the program implementation time in order to examine the longer-term learning effects of these pedagogical models.

## Conclusion

The findings of this study were that the TGfU-SE model had more positive learning effects on students’ learning motivation, sport enjoyment, responsibility, and game performance than the TGfU model alone. Thus, the conclusion was that the TGfU-SE model had a stronger positive influence on students’ learning effects in PE lessons than the TGfU model alone. The findings of this research will contribute to research development for model-based practice in PE. Integrating TGfU with the SE model not only develops students’ tactical awareness and motor skills but also develops positive affective behaviors over a game season. Students experienced group affiliation and individual duty in their own team in the TGfU-SE model. PE teachers should enable students to do their best on their own team in PE lessons. In addition, students can cooperate with each other to obtain improved performance in games. Integrating TGfU with the SE model can promote all components of the cognitive, psychomotor, and affective domains. Based on the results of the study, we suggest that schools hold teaching workshops for PE teachers to learn how to integrate TGfU into the SE model to improve the learning effect in PE lessons. In addition, the TGfU, SE, and TGfU-SE models should be incorporated into pre-service PE teacher education programs to develop pre-service PE teachers’ professional competence in PE teaching. In terms of suggestions for future research, future studies should include a broader sample of teachers and students, as well as a greater variety in terms of the sports played and different age levels. Second, the teaching time in this study should be extended to examine whether the learning effects improve more significantly between the experimental and control groups. Third, a long-term longitudinal study should be conducted to gain an in-depth understanding of the hybrid TGfU-SE model.

## Data availability statement

The original contributions presented in the study are included in the article/supplementary material, further inquiries can be directed to the corresponding author.

## Ethics statement

The studies involving human participants were reviewed and approved by Institutional Review Board, Fu Jen Catholic University. Written informed consent to participate in this study was provided by the participants’ legal guardian/next of kin.

## Author contributions

Y-HP contributed in this study about literature review, research design, result, and discussion. C-HH contributed in this study about research design, statistic analysis, questionaire survey, result, and discussion. W-TH contributed in this study about literature review, research design, result, and discussion. All authors contributed to the article and approved the submitted version.

## Funding

This work was supported by the Ministry of Science and Technology, R.O.C. under Grant MOST 105-2410-H-179-005.

## Conflict of interest

The authors declare that the research was conducted in the absence of any commercial or financial relationships that could be construed as a potential conflict of interest.

## Publisher’s note

All claims expressed in this article are solely those of the authors and do not necessarily represent those of their affiliated organizations, or those of the publisher, the editors and the reviewers. Any product that may be evaluated in this article, or claim that may be made by its manufacturer, is not guaranteed or endorsed by the publisher.
